# A UPF0118 family protein with uncharacterized function from the moderate halophile *Halobacillus andaensis* represents a novel class of Na^+^(Li^+^)/H^+^ antiporter

**DOI:** 10.1038/srep45936

**Published:** 2017-04-04

**Authors:** Ping Dong, Lidan Wang, Na Song, Lina Yang, Jin Chen, Mingxue Yan, Huiwen Chen, Rui Zhang, Jincheng Li, Heba Abdel-motaal, Juquan Jiang

**Affiliations:** 1 Department of Microbiology and Biotechnology, College of Life Sciences, Northeast Agricultural University, Harbin, 150030, PR China

## Abstract

In this study, genomic DNA was screened from *Halobacillus andaensis* NEAU-ST10-40^T^ by selection in *Escherichia coli* KNabc lacking three major Na^+^/H^+^ antiporters. One gene designated *upf0118* exhibiting Na^+^(Li^+^)/H^+^ antiport activity was finally cloned. Protein alignment showed that UPF0118 shares the highest identity of 81.5% with an unannotated gene encoding a protein with uncharacterized protein function belonging to UPF0118 family from *H. kuroshimensis*, but shares no identity with all known specific Na^+^(Li^+^)/H^+^ antiporter genes or genes with Na^+^(Li^+^)/H^+^ antiport activity. Growth test, western blot and Na^+^(Li^+^)/H^+^ antiport assay revealed that UPF0118 as a transmembrane protein exhibits pH-dependent Na^+^(Li^+^)/H^+^ antiport activity. Phylogenetic analysis indicated that UPF0118 clustered with all its homologs belonging to UPF0118 family at a wide range of 22–82% identities with the bootstrap value of 92%, which was significantly distant with all known specific single-gene Na^+^(Li^+^)/H^+^ antiporters and single-gene proteins with the Na^+^(Li^+^)/H^+^ antiport activity. Taken together, we propose that UPF0118 should represent a novel class of Na^+^(Li^+^)/H^+^ antiporter. To the best of our knowledge, this is the first report on the functional analysis of a protein with uncharacterized protein function as a representative of UPF0118 family containing the domain of unknown function, DUF20.

In prokaryotes, Na^+^/H^+^ antiporters are ubiquitous secondary transporters catalyze the efflux of intracellular alkali cations in exchange for external protons, which play a vital role in reducing the cytoplasmic concentration of toxic alkali cations and supporting Na^+^-dependent intracellular pH homeostasis under alkaline conditions^1^,^2^,^3^,^4^. They were also designated Na^+^(Li^+^)/H^+^ antiporters, due to Na^+^/H^+^ antiport activity, together with Li^+^/H^+^ antiport activity. Since the gene *ant* was found for the first time to affect the Na^+^/H^+^ antiport activity of *Escherichia coli* and therefore designated *nhaA*^5^, Na^+^(Li^+^)/H^+^ antiporter genes or the genes with Na^+^(Li^+^)/H^+^ antiport activity have been increasingly cloned and functionally identified in *E. coli* mutants KNabc or EP432, which lack major antiporters^6^,^7^. In present, Na^+^(Li^+^)/H^+^ antiporters are divided into two main categories based on the number of encoding genes: one category of Na^+^(Li^+^)/H^+^ antiporters are encoded by a single gene such as *nhaA*^6^,^8^, *nhaB*^9^,^10^, *nhaC*^11^, *nhaD*^8^,^11^,^12^,^13^,^14^,^15^,^16^, *nhaE*^17^, *napA*^18^, *nhaP*^19^, *nhaG*^20^ or *nhaH*^21^, all of which are grouped into the monovalent Cation/Proton Antiporter 1 (CPA-1) family with the exception of NapA sharing the high identity with K^+^/H^+^ antiporters that are grouped into the CPA-2 family^22^. The other category containing six or seven subunits are encoded by a multi-cistronic operon with the different designations such as *mrp*^2^,^23^,^24^,^25^, *mnh*^26^, *pha*^27^,^28^,^29^,^30^ or *sha*^31^, which are grouped into the CPA-3 family due to its distinctive multigene structural properties^22^. In addition to two above mentioned main categories, other genes with Na^+^(Li^+^)/H^+^ antiport activity were also continually shown to exhibit Na^+^/H^+^ antiport activity. For example, ChaA was previously reported to have properties of a Ca^2+^/H^+^ antiporter and a Na^+^/H^+^ antiporter^31^, and was later characterized to also be a K^+^/H^+^ antiporter^32^. MleN was identified to be a malic^−2^-2H^+^/Na^+^-lactate^−1^ antiporter, which exhibits Na^+^/H^+^ antiport activity coupled with malate/lactate antiport activity^33^. An unique tetracycline/H^+^ antiporter TetB(L)^34^ and a primary Na^+^ pump Nap^35^ were reported to possess Na^+^/H^+^ antiport activity. The *E. coli* multidrug resistance (MDR) protein MdfA with a broad-specificity MDR phenotype^36^ was also characterized to exhibit Na^+^(K^+^)/H^+^ antiport activity^37^. Putative paired small multidrug resistance family proteins PsmrAB, the homolog of YvdSR, were characterized to function mainly as a novel two-component Na^+^/H^+^ antiporter^38^.

In our recent study^39^, strain NEAU-ST10-40^T^, a moderate halophile isolated from Na_2_CO_3_-type saline and alkaline soils in Anda City, China was identified to represent a novel species *Halobacillus, Halobacillus andaensis*, with the growth range of NaCl concentrations of 3–15% (w/v) (optimum, 8%, w/v) and pH 7.0–9.0 (optimum, pH 8.0), and thus this novel halophilic strain from unique saline-alkaline conditions could have developed sophisticated mechanisms to maintain its intracellular steady osmotic and ionic state. Since almost all halophilic microorganisms have the ability to expel Na^+^ from the interior of the cells using Na^+^(Li^+^)/H^+^ antiporters^40^,^41^, it is very likely that important (even novel) Na^+^(Li^+^)/H^+^ antiporter genes exist in this novel strain NEAU-ST10-40^T^, a moderate halophile which can tolerate up to 15% (w/v) NaCl.

In order to obtain as many (especially novel) Na^+^(Li^+^)/H^+^ antiporter genes as possible, genomic DNA was screened from *H. andaensis* NEAU-ST10-40^T^ for the Na^+^(Li^+^)/H^+^ antiporter gene by selection in *E. coli* KNabc lacking three major Na^+^(Li^+^)/H^+^ antiporters. Of several screened resultant clones, to our surprise, one gene designated *upf0118* showed the highest identity of 81.5% with an unannotated gene encoding a protein with uncharacterized protein function belonging to UPF0118 family from *H. kuroshimensis*, but showed no identity with all known specific Na^+^(Li^+^)/H^+^ antiporter genes or genes with Na^+^(Li^+^)/H^+^ antiport activity. In this study, we reported the cloning and characterization of *upf0118* and propose that this novel protein belonging to UPF0118 family should represents a novel class of Na^+^(Li^+^)/H^+^ antiporter.

## Results

### Cloning and sequence analysis of the gene with Na^+^(Li^+^)/H^+^ antiport activity

**Figure 1:**
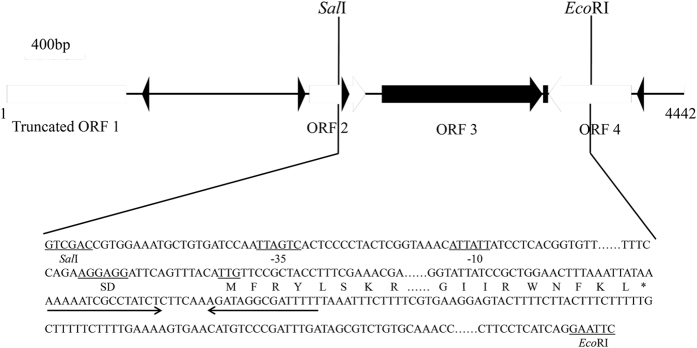
The mapping of the inserted DNA fragment in the recombinant plasmid pUC-DP61 and subcloning strategy of the gene *upf0118*. One C-terminus truncated ORF and three intact ORFs are included in a 4.4-kb DNA fragment inserted into the recombinant plasmid pUC-DP61, each of which is preceded by a respective promoter-like sequence and a respective Shine-Dalgarno (SD) sequence. A predicted promoter sequence (−35 region and −10 region) and Shine-Dalgarno (SD) sequence, the initiation codon TTG of ORF3 designated UPF0118 (Genbank accession No. KY231907), the enzymatic site of *Sal*I and *Eco*RI are underlined. The stop codon TAA of ORF3 is indicated by the asterisk and a possible terminator following ORF3 indicated by inverted solid arrows. UPF0118 was subcloned, together with its promoter-like and SD sequences, into *Sal*I- and *Eco*RI-digested pUC18 through digestion by the same two restriction enzymes and ligation.

As shown in Fig. 1, a 4.4-kb DNA fragment was obtained from *Sau*3AI-digested genomic DNA from strain NEAU-ST10-40^T^ using *E. coli* KNabc. The recombinant plasmid designated pUC-DP61 (pUC18 carrying this DNA fragment) enabled *E. coli* KNabc cells to grow on the LBK plate containing 0.2 M NaCl. Sequence analysis showed that one C-terminus truncated ORF (ORF1) and three intact ORFs (ORF2-4) are included in this DNA fragment, each of which is preceded by a respective promoter-like sequence and a respective Shine-Dalgarno (SD) sequence (Fig. 1). C-terminus truncated ORF1 has the highest identity of 76.0% with a putative glycine betaine transporter (accession.version No. EKE30543.1) from *Salimicrobium jeotgali*, ORF2 has the highest identity of 73.0% with a hypothetical protein (accession.version No. WP_ 026577524.1) belonging to YugN-like family from an uncharacterized *Bacillaceae* species, ORF3 has the highest identity of 81.5% with a protein (accession.version No. WP_027954082.1, the corresponding gene is unannotated in its genome) belonging to UPF0118 family with uncharacterized protein function from *H. kuroshimensis*, and ORF4 has the highest identity of 63.0% with a putative N-acetyltransferase (accession.version No. WP_035545378.1) from *Halobacillus* sp. BBL2006.

**Figure 2:**
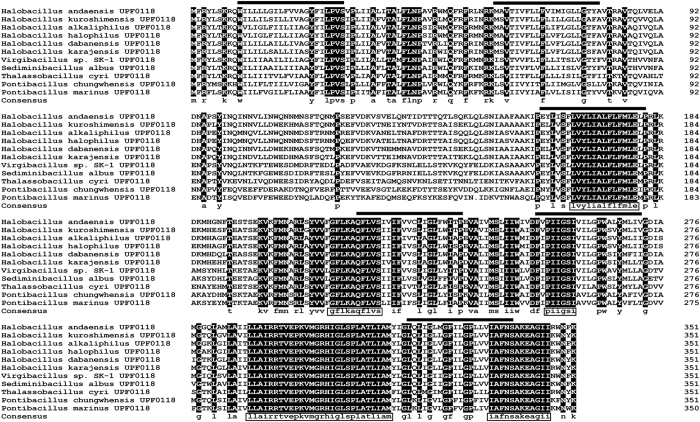
Alignment of UPF0118 with its most closely related holomogs clustered within the neighbour-joining phylogenetic tree. The ten closest homologs with 58–82% identities clustered within the neighbour-joining phylogenetic tree with the bootstrap value of 100% (Fig. 3) were selected to show the conserved motifs and amino acid residues. Acession.version numbers are shown in the neighbour-joining phylogenetic tree of Fig. 3. Shading homology corresponds to 100% (black), >75% (grey), ≥50% (lightgrey) and <50% (white) amino acid identity, respectively. The six putative transmembrane segments are marked with bold lines above the alignment. The five highly conserved motifs are highlighted with the open rectangles in the consensus sequence.

Among the above mentioned four ORFs, ORF1 is incomplete and therefore can’t restore the growth of *E. coli* KNabc in the presence of 0.2 M NaCl. As for the other three intact ORFs, ORF3, an uncharacterized function protein belonging to UPF0118 family, is predicted to be the sole transmembrane protein composed of six putative transmembrane segments (TMSs) including TMS I (10–30), TMS II (60–80), TMS III (161–181), TMS IV (217–237), TMS V (252–272), TMS VI (319–339) (Fig. 2). Hereafter, the gene encoding this protein was designated *upf0118* in order to describe the following identification. The deduced amino acid sequence of UPF0118 consists of 352 residues with a calculated molecular weight of 39, 611.01 Dalton and a pI of 9.76. Among the 352 residues of UPF0118, 225 residues were hydrophobic, indicating that UPF0118 is of low polarity. Based on the above sequence analysis, ORF3 is the most likely to exhibit Na^+^(Li^+^)/H^+^ antiport activity, since Na^+^(Li^+^)/H^+^ antiporters must be transmembrane proteins of low polarity.

### Identification of ORFs with Na^+^(Li^+^)/H^+^ antiport activity

In order to identify the exact ORF with Na^+^(Li^+^)/H^+^ antiport activity, UPF0118 with its promoter-like and SD sequences was at first subcloned into *Sal*I- and *Eco*RI-digested pUC18 through digestion by the same two restriction enzymes and ligation (Fig. 1) and chemically transformed into *E. coli* KNabc to test if its presence could restore the growth of *E. coli* KNabc in the presence of 0.2 M NaCl. Also, ORF2 or ORF4 was tested to check whether either of them could restore the growth of *E. coli* KNabc in the presence of 0.2 M NaCl, respectively, through the similar methods using the suitable restriction enzymatic digestion, ligation and transformation. As a result, the expression of only *upf0118*, but ORF2 or ORF4 not, enabled *E. coli* KNabc to grow in the presence of 0.2 M NaCl. Therefore, UPF0118 is exactly likely to possess Na^+^(Li^+^)/H^+^ antiport activity. The resultant plasmid containing subcloned *upf0118* with its promoter-like and SD sequences was therefore designated pUC-UPF0118.

Phylogenetic analysis and protein alignment between UPF0118 and its homologs, together with identified specific single-gene Na^+^(Li^+^)/H^+^ antiporters and single-gene proteins with Na^+^(Li^+^)/H^+^ antiport activity.

**Figure 3:**
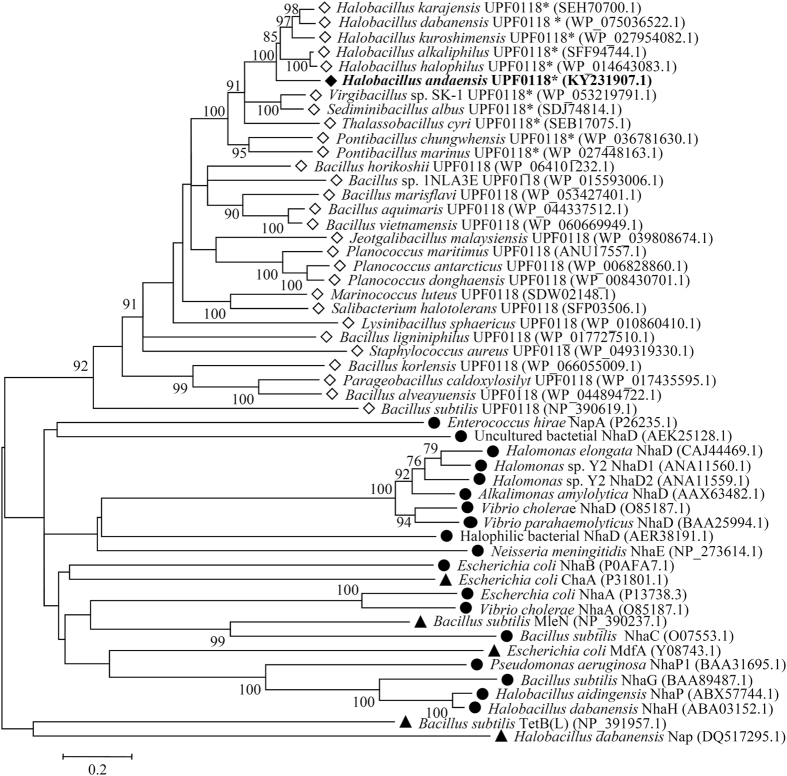
Neighbour-joining phylogenetic tree of selected homologs of *Halobacillus andaensis* UPF0118, together with known Na^+^/H^+^ antiporters. For the construction of phylogenetic tree, nine closest homologs with 60–82% identities, nine closer homologs with 49–59% identities, nine distant homologs with 22–42% identities, together with all representatives of known specific single-gene Na^+^/H^+^ antiporters and single-gene proteins with the Na^+^/H^+^ antiport activity were selected. Accession.version numbers of selected homologs were shown in the parenthesis. Open diamond stands for putative UPF0118 family proteins; filled diamond stands for UPF0118 identified in this study; filled circle stands for known specific single-gene Na^+^/H^+^ antiporters; filled triangle stands for other single-gene proteins with the Na^+^/H^+^ antiport activity. Bootstrap values ≥70% (based on 1000 replications) are shown at branch points. Bar, 0.2 substitutions per amino acid residue position. UPF0118 and its most closely related holomogs clustered with the bootstrap value of 99% marked with the asterisk were used for protein alignment in Fig. 2.

To show whether this novel protein indeed belongs to UPF0118 family and whether it shares phylogenetic relationship with identified specific single-gene Na^+^(Li^+^)/H^+^ antiporters and other single-gene proteins with Na^+^(Li^+^)/H^+^ antiport activity, phylogenetic analysis based on neighbour-joining algorithm was carried out. For the construction of phylogenetic tree, nine closest homologs with 60–82% identities, nine closer homologs with 49–59% identities and nine distant homologs with 22–42% identities, together with all representatives of known specific single-gene Na^+^/H^+^ antiporters and other single-gene proteins with the Na^+^/H^+^ antiport activity were selected. As shown in Fig. 3, UPF0118 clustered with all its homologs belonging to UPF0118 family with the bootstrap value of 92%, which was significantly distant with all known specific single-gene Na^+^(Li^+^)/H^+^ antiporters and single-gene proteins with the Na^+^(Li^+^)/H^+^ antiport activity.

UPF0118 was also aligned with its ten closest homologs clustered within the neighbour-joining phylogenetic tree with the bootstrap value of 100%. In addition to the homolog with the highest identity of 81.5% from *H. kuroshimensis*, UPF0118 has the respective identities of 79.3%, 78.7%, 78.7%, 77.8%, 69.0%, 67.3%, 65.1%, 63.1% and 58.4% with the homologs from *H. alkaliphilus* (accession.version No. SFF94744.1), *H. halophilus* (accession.version No. WP_014643083.1), *H. dabanensis* (accession.version No. WP_075036522.1), *H. karajensis* (accession.version No. SEH70700.1), *Virgibacillus* sp. SK-1 (accession.version No. WP_053219791.1), *Sediminibacillus albus* (accession.version No. SDJ74814.1), *Thalassobacillus cyri* (accession.version No. SEB17075.1), *Pontibacillus chungwhensis* (accession.version No. WP_036781630.1) and *P. marinus* (accession.version No. WP_027448163.1) (Fig. 2). At a wide range of 58–82% identities, five highly conserved motifs including “LVYLIALFLFMLE”, “GFLKAQFLVS”, “PIIGSI”, “LLAIRRTVEPKVMGRHIGLSPLATLIAM” and “IAFNSAKEAGII” were found among all the selected homologs (Fig. 2). It should be noted that UPF0118 was also aligned with all known specific Na^+^(Li^+^)/H^+^ antiporters and proteins with the Na^+^(Li^+^)/H^+^ antiport activity, but showed no identity with each of them.

### Resistance of UPF0118 to NaCl, LiCl and pH

**Figure 4:**
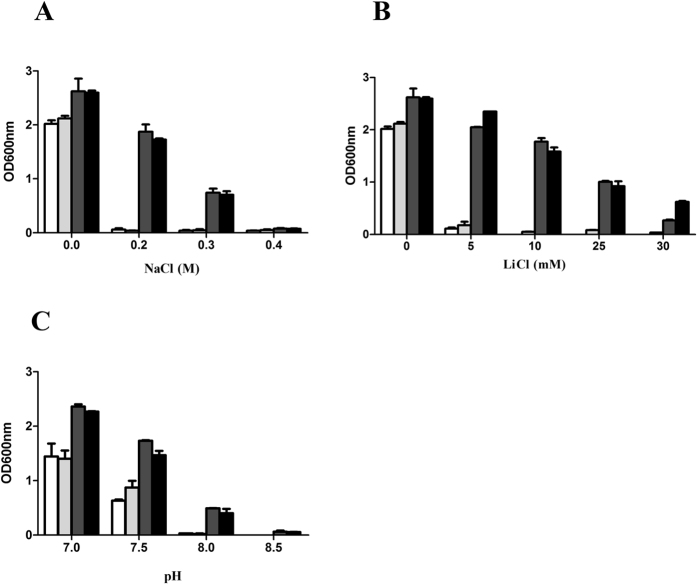
Salt tolerance and alkaline pH resistance of *E. coli* strains KNabc. For the salt tolerance test, 1% overnight cultures of KNabc transformant cells carrying the empty vector pUC18 (white column), pET-22b (lightgrey column), pUC-UPF0118 (darkgrey column) or pET-22b-UPF0118 (black column) grown at 37 °C in the LBK medium at pH 7.0 were innoculated into fresh LBK medium at pH 7.0, to which NaCl (**A**) or LiCl (**B**) was added at indicated concentrations, followed by incubation at 37 °C. To test the effect of pH (**C**) on cell growth, 1% overnight cultures of the above mentioned KNabc transformant cells grown at 37 °C in the LBK medium at pH 7.0 were innoculated into fresh LBK medium containing 50 mM NaCl at indicated pHs adjusted by adding the Hepes-Tris buffer at the final concentration of 100 mM, followed by incubation at 37 °C. The above mentioned cell growth was ended after 24 h and monitored turbidimetrically at 600 nm. Each data point represents the average of three independent determinations.

To test the ability of UPF0118 to induce salt tolerance, *E. coli* KNabc/pUC-UPF0118 and KNabc/pUC18 were grown in LBK medium containing 0–0.4 M NaCl or 0–30 mM LiCl. As shown in Fig. 4A, *E. coli* KNabc/pUC-UPF0118 could grow in the presence of 0.3 M NaCl, but *E. coli* KNabc/pUC18 as a negative control could not survive in the presence of 0.2 M NaCl. Also, *E. coli* KNabc/pUC-UPF0118 could grow only in the presence of 30 mM LiCl, but *E. coli* KNabc/pUC18 as a negative control could not survive in the presence of 5 mM LiCl (Fig. 4B). In order to analyze the resistance of UPF0118 to alkaline pH, *E. coli* KNabc/pUC-UPF0118 and KNabc/pUC18 were grown in the LBK medium at the pH values from 7.0 to 8.5. As shown in Fig. 4C, the growth of *E. coli* KNabc/pUC18 was greatly reduced under alkaline conditions, especially at pH 8.0, compared with that at pH 7.0, whereas the expression of *upf0118* conferred *E. coli* KNabc cells the capability to grow under alkaline conditions. For the more detailed analysis of the resistance of UPF0118 to NaCl, LiCl and pH, the growth cuvres of *E. coli* KNabc/pUC-UPF0118 and KNabc/pUC18 in the presence of 0.2 M NaCl or 5 mM LiCl, or at pH 7.0, pH 7.5 were plotted on a semilogarithmic scale. As shown in Fig. S1, the expression of *upf0118* conferred *E. coli* KNabc cells the significant tolerance to 0.2 M NaCl or 5 mM LiCl, and an alkline pH resistance at 7.5.

### Detection and localization of UPF0118 by western blot

**Figure 5:**
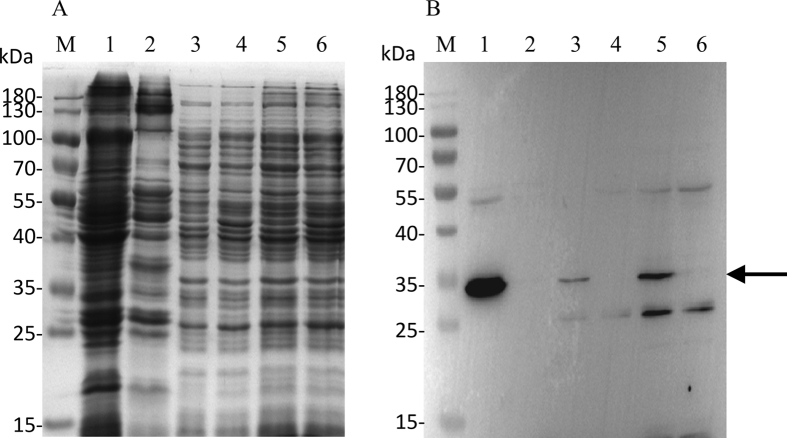
Western blot detection and localization of UPF0118 in *Escherichia coli*. For the detection and localization of UPF0118, *E. coli* KNabc carrying pET-22b-UPF0118 and the empty vector pET-22b (as a negative control) were grown in LBK medium to OD_600_ between 0.4 and 0.6 at 37 °C, followed by induction by the addition of isopropyl-β-D-thiogalactoside to a final concentration of 1 mM at 28 °C for an additional 6 h and then harvested by centrifugation at 5, 000 g, 4 °C for 10 min and washed three times with Tris-HCl (10 mM Tris -HCl, pH 7.5). The membrane protein fractions, cytoplasmic protein ones and total protein extract from *E. coli* KNabc/pET-22b-UPF0118 (Lanes 1, 3, 5) and KNabc/pET-22b (Lanes 2, 4, 6) were sampled, respectively, and then used for SDS-PAGE (**A**) and western blots (**B**). The position of target protein UPF0118 fused with a N-terminal His_6_ tag is shown with a solid arrow.

For the detection and localization of UPF0118, the expression vector pET-22b-UPF0118 was constructed and the sole ORF of *upf0118* gene was fused in frame with an N-terminal His_6_ tag. The growth test showed that *E. coli* KNabc/pET-22b-UPF0118 exhibited the similar tolerance to the salts and alkaline pH resistance (Fig. 4), compared with KNabc/pUC-UPF0118. Also, sequencing analysis revealed that UPF0118 succeeded in being fused in frame with an N-terminal His_6_ tag. Therefore, pET-22b-UPF0118 can be used for the following western blot experiments. As shown in Fig. 5, the expression of UPF0118 was detected in the total protein extract, membrane protein fractions and cytoplasmic protein ones from *E. coli* KNabc/pET-22b-UPF0118, but not in those from KNabc/pET-22b.

### Na^+^(Li^+^)/H^+^ antiport activity in everted membrane vesicles

**Figure 6:**
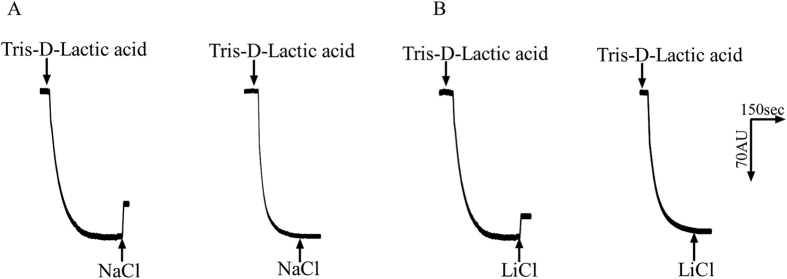
Assays for the Na^+^(Li^+^)/H^+^ antiport activity in the everted membranes. The activity measurements for Na^+^/H^+^ antiporter (**A**), Li^+^/H^+^ antiporter (**B**) were performed at pH 9.0 in everted membrane vesicles prepared from cells of *E. coli* KNabc/pUC-UPF0118 (to the left) or KNabc/pUC18 (to the right) by the French pressure cell method. At the time points indicated by downward arrows, Tris-D-lactic acid (final concentration 10 mM) was added to the assay mixture to initiate fluorescence quenching. At the time points indicated by upward arrows, NaCl (final concentration 10 mM) or LiCl (final concentration 10 mM) was added to the assay mixture. Fluorescence quenching is shown in arbitrary units.

**Figure 7:**
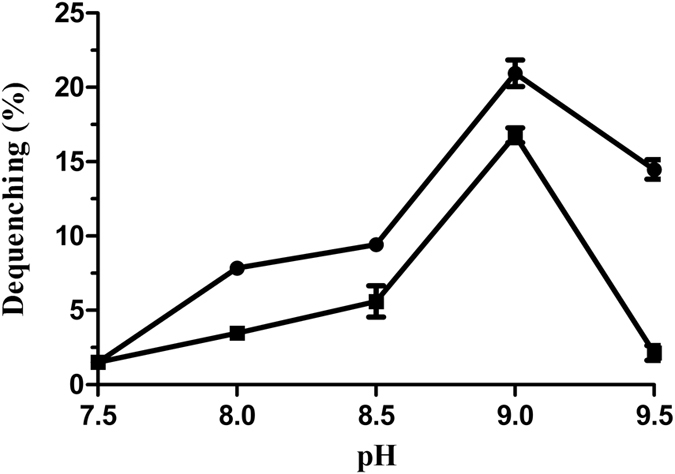
pH-dependent profile of the Na^+^(Li^+^)/H^+^ antiport activity of UPF0118. The antiporter activity was measured by the fluorescence quenching method. Na^+^/H^+^ antiport activity (filled circle) and Li^+^/H^+^ antiport activity (filled square) were measured at the indicated pH adjusted by 10 mM BTP buffer. The wavelength of excitation light was 492 nm and fluorescence was monitored at 526 nm. Each value point represents the average of three independent determinations.

Na^+^(Li^+^)/H^+^ antiport activity with everted membrane vesicles prepared from cells of *E. coli* KNabc strains carrying pUC-UPF0118 or pUC18 was determined by measuring the dequenching of acridine orange fluorescence upon addition of NaCl or LiCl. As shown in Fig. 6, both Na^+^/H^+^ and Li^+^/H^+^ antiport activity were detected in everted membrane vesicles from KNabc/pUC-UPF0118, while no Na^+^/H^+^ or Li^+^/H^+^ antiport activity was detected in those from KNabc/pUC18. The effect of pH on Na^+^/H^+^ as well as Li^+^/H^+^ antiport activity was also measured. UPF0118 exhibited Na^+^/H^+^ or Li^+^/H^+^ antiport activity at a wide range of pH between 7.5 and 9.5, with the highest Na^+^/H^+^ and Li^+^/H^+^ antiport activity at pH 9.0 (Fig. 7). Also, K^+^/H^+^ antiport activity was measured in everted membrane vesicles from KNabc/pUC-UPF0118, but no activity was detected.

**Figure 8:**
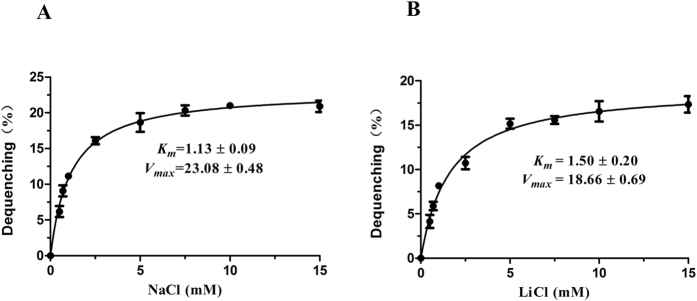
Michaelis-Menten kinetics and calculation of the apparent *K*_*m*_ and *V*_*max*_ values of UPF0118. Na^+^/H^+^ (**A**) and Li^+^/H^+^ (**B**) antiport activity of UPF0118 were plotted as the respective function of cation concentrations. For the calculation of the apparent *K*_*m*_ and *V*_*max*_ values of UPF0118, non-linear regression analysis was carried out and the corresponding apparent *K*_*m*_ and *V*_*max*_ values were obtained with Prism 5.0, respectively. Each value point represents the average of three independent determinations.

In order to assess the affinity and maximal velocity of UPF0118 for Na^+^ or Li^+^, Michaelis-Menten kinetics of UPF0118 for Na^+^ and Li^+^ were analyzed, respectively, by measuring Na^+^/H^+^ and Li^+^/H^+^ antiport activity in everted membrane vesicles from KNabc/pUC-UPF0118 at pH 9.0 with final concentrations of added NaCl or LiCl varying from 0–15 mM. The apparent *K*_*m*_ and *V*_*max*_ values of UPF0118 for Na^+^ and Li^+^ were finally calculated to be 1.13 ± 0.09 mM/23.08 ± 0.48 mM (Fig. 8A) and 1.50 ± 0.20 mM/18.66 ± 0.69 mM (Fig. 8B), respectively. This suggests that Na^+^ is a better substrate than Li^+^ for the antiporter.

## Discussion

In this study, we showed a protein with uncharacterized function, UPF0118, from the moderate halophile NEAU-ST10-40^T^ exhibits Na^+^(Li^+^)/H^+^ antiport activity, whose homologs have not been annotated in their genomes as yet. Based on the protein identity and phylogenetic analysis, UPF0118 should represent a novel class of Na^+^(Li^+^)/H^+^ antiporter, which is significantly different from all known single-gene Na^+^(Li^+^)/H^+^ antiporters^5^,^8^,^9^,^10^,^11^,^12^,^13^,^14^,^15^,^16^,^17^,^18^,^19^,^20^,^21^ or single-gene proteins with the Na^+^(Li^+^)/H^+^ antiport activity^32^,^33^,^34^,^35^,^42^. To the best of our knowledge, this is the first report on the functional analysis of a protein with uncharacterized function as a representative of UPF0118 family.

UPF0118 was predicted to contain six putative transmembrane segments (Fig. 2), which was confirmed by the result that UPF0118 was mainly localized in the membrane protein fractions from *E. coli* KNabc/pET-22b-UPF0118 (Fig. 5). However, a weak signal was also detected in the cytoplasmic protein fractions from *E. coli* KNabc/pET-22b-UPF0118 (Fig. 5). We speculated that the membrane protein fractions could not be absolutely separated from the cytoplasmic protein ones by ultracentrifugation, which may lead to a trace amount of the former remaining in the latter. Combined with hydrophobic analysis of UPF0118, UPF0118 is a transmembrane protein of low polarity, which is consistent with the reports on Na^+^(Li^+^)/H^+^ antiporters^1^,^2^,^3^,^4^. UPF0118 could confer the *E. coli* KNabc the salt tolerance of 0.3 M NaCl and 30 mM LiCl, and the capability of growing under alkaline conditions (Fig. 4). The pH-dependent Na^+^/H^+^ and Li^+^/H^+^ antiport activity was detected in everted membrane vesicles from KNabc/pUC-UPF0118 at a wide pH range from 7.5 to 9.5, with the highest Na^+^/H^+^ antiport and Li^+^/H^+^ antiport activity at pH 9.0 (Fig. 7), but not from KNabc/pUC18 (Fig. 6). Therefore, UPF0118 is likely to encode a pH-dependent Na^+^(Li^+^)/H^+^ antiporter, which is consistent with the reports on Na^+^(Li^+^)/H^+^ antiporters^1^,^2^,^3^,^4^,^8^. Also, UPF0118 exhibited Na^+^(Li^+^)/H^+^ antiport activity mainly under alkaline conditions from pH 8.0 to 9.5 (Fig. 7), which may be related to the adaptation of its host to the extremophilic saline-alkaline conditions, since strain NEAU-ST10–40^T^ is a moderate halophile with the optimum growth at the 8% (w/v) NaCl concentrations and at pH 8.0^39^.

A careful protein alignment using BLASTp at the NCBI website^43^ revealed that UPF0118 shares no identity with all representatives of known specific single-gene Na^+^(Li^+^)/H^+^ antiporters and single-gene proteins with the Na^+^(Li^+^)/H^+^ antiport activity, even putative Na^+^(Li^+^)/H^+^ antiporters. This is confirmed with the phylogenetic analysis of selected homologs of UPF0118 at a wide range of 22–82% identities, together with all known specific single-gene Na^+^(Li^+^)/H^+^ antiporters and single-gene proteins with the Na^+^(Li^+^)/H^+^ antiport activity (Fig. 3). Therefore, we propose that UPF0118 should represent a novel class of Na^+^/H^+^ antiporter.

UPF0118 family proteins are one category of predicted transmembrane proteins with uncharacterized protein function containing a conserved domain of unknown function (designated DUF20, a protein sequence homologous to the one corresponding to UPF0118 from No. 10 residue to No. 346 residue containing six TMSs)^44^. Based on the protein alignment between UPF01118 and its ten closest homologs, five highly conserved motifs, especially “LLAIRRTVEPKVMGRHIGLSPLATLIAM”, were found among the selected homologs at a wide range of 58–82% identities. Even though the homologs of UPF0118 were broaden to a wider range of 30–82% identities, this motif is also relatively conserved with the exception of several residues. In the future study, we plan to delete this motif, or each of other four ones, or replace the conserved acidic or polar residues in the domain of unknown function, DUF20, to identify whether they are involved in the Na^+^(Li^+^)/H^+^ antiport activity. Since UPF0118 and its selected homologs clustered with the bootstrap value of 92%, we propose that the function of UPF0118 should represent those of this family proteins. As shown at the website https://www.ncbi.nlm.nih.gov/Structure/cdd/wrpsb.cgi, no information suggested the exact function of UPF0118 family proteins with the domain of unknown function, DUF20. In this study, we at least showed that UPF0118 exhibits Na^+^(Li^+^)/H^+^ antiport activity, which is the first report on the functional analysis of a protein with uncharacterized protein function as a representative of UPF0118. The results presented in this manuscript triggers the understanding of the function of UPF0118 family proteins containing the domain of unknown function, DUF20.

## Materials and Methods

### Bacterial strains and culture conditions

*H. andaensis* NEAU-ST10-40^T^ was grown in 8% (w/v, optimum) NaCl modified S-G liquid medium at pH 8.0 (optimum) with the following composition: 1.0% tryptone, 0.5% yeast extract, 0.5% casein, 0.2% KCl, 0.3% sodium citrate, 2.0% MgSO_4_ · 7H_2_O, 8.0% NaCl, as described by Wang *et al*.^39^. *E. coli* strain KNabc, lacking three major Na^+^/H^+^ antiporters (NhaA, NhaB and ChaA)^6^ and its transformant cells carrying either the empty vector pUC18, pET-22b, or the recombinant plasmids pUC-DP61 (pUC18 carrying a 4.4-kb DNA fragment with Na^+^/H^+^ antiport activity), pUC-UPF0118 (pUC18 carrying UPF0118 only) or pET-22b-UPF0118 (pET-22b carrying UPF0118 fused with an N-terminal His_6_ tag) were grown in the LBK medium consisting of 1.0% tryptone, 0.5% yeast extract, and 87 mM KCl as described by Karpel *et al*.^5^. For the salt tolerance test, 1% overnight cultures of KNabc transformant cells grown at 37 °C in the LBK medium at pH 7.0 were innoculated into fresh LBK medium at pH 7.0, to which NaCl or LiCl was added at indicated concentrations, followed by incubation at 37 °C. To test the effect of pH on cell growth, the KNabc transformant cells grown at 37 °C in the LBK medium at pH 7.0 were innoculated into fresh LBK medium containing 50 mM NaCl at indicated pHs adjusted by adding the Hepes-Tris buffer to a final concentration of 100 mM, followed by incubation at 37 °C. It should be pointed out that Na^+^(Li^+^)/H^+^ antiporters can offer alkaline pH resistance only in the presence of Na^+^ or Li^+^. Therefore, a certain amount of Na^+^ such as 50 mM NaCl needs be added to the tested medium^1^,^2^,^3^,^4^. The above mentioned cell growth was ended after 24 h and monitored turbidimetrically at 600 nm. For the more detailed analysis of the resistance of UPF0118 to NaCl, LiCl and pH, 1% overnight cultures of KNabc cells carrying the empty vector pUC18 or pUC-UPF0118 grown at 37 °C in the LBK medium at pH 7.0 were innoculated into fresh LBK medium at pH 7.0, to which 0. 2 M NaCl or 5 mM LiCl was added, or fresh LBK medium containing 50 mM NaCl at pH 7.5 adjusted by adding the Hepes-Tris buffer at the final concentration of 100 mM, followed by incubation at 37 °C. Also, to show cell growth in the absence of the salts at neutral pH, the KNabc transformant cells carrying the empty vector pUC18 or pUC-UPF0118 grown at 37 °C in the LBK medium at pH 7.0 were innoculated into fresh LBK medium without the addition of the tested salts at pH 7.0 adjusted by adding the Hepes-Tris buffer at the final concentration of 100 mM, followed by incubation at 37 °C. The above mentioned cell growth was monitored turbidimetrically at 600 nm at the indicated time points within 28 h. The growth cuvres were plotted on a semilogarithmic scale. Ampicillin was added to a final concentration of 50 μg · ml^−1^ for the selection and growth of cells containing plasmids. Eletrocompetent *E. coli* cells were prepared and electroporated according to the protocol described in our previous study^38^.

### Screening of the DNA fragment containing Na^+^/H^+^ antiporter gene and subcloning of UPF0118

The genomic DNA was extracted from strain NEAU-ST10-40^T^, and partially digested with *Sau*3AI. The DNA fragments with 4–10 kb were separated by agarose electrophoresis and ligated into pUC18, which had been digested with *Bam*HI and dephosphorylated with bacterial alkaline phosphatase, using T4 DNA ligase. Electrocompetent cells of *E. coli* KNabc were transformed with the ligated reaction mixture and spread on LBK plates containing 0.2 M NaCl, 1.5% agar and 50 μg·ml^−1^ of ampicillin. The plates were incubated at 37 °C for 24 h and colonies picked for further studies. For the identification of the exact ORF with Na^+^(Li^+^)/H^+^ antiport activity, UPF0118 was subcloned, together with its promoter-like and SD sequences, into *Sal*I- and *Eco*RI-digested pUC18 through digestion by the same two restriction enzymes and ligation (Fig. 1). Also, subcloning strategy of ORF2 or ORF4 was similar to that of UPF0118. For the detection and localization of UPF0118, the sole ORF of *upf0118* gene was fused in frame with an N-terminal His_6_ tag followed by a thrombin proteolytic cleavage site and a T7 tag in an expression vector pET-28b (Novagen Co. Ltd) through PCR amplification, restriction enzyme digestion and ligation. The forward primer is 5′-GGATCCGATGTTCCGCTACCTTTCG-3′ (*Bam*HI underlined) and the reverse primer is 5′-CTCGAGTTATAATTTAAAGTTCCAG-3′ (*Xho*I underlined). Because *E. coli* KNabc and vector pET-28b carry kanamycin resistance as a selection indicator, the resultant plasmid designated pET28b-UPF0118, which can’t be used for the complementation with *E. coli* KNabc, was double-digested by using *Bgl*II and *Xho*I, and then the smaller band was separated by agarose electrophoresis and ligated into an expression vector pET-22b (Novagen Co. Ltd), with an ampcillin resistance as a selection indicator. The resultant expression construct designated pET-22b-UPF0118 was verified by sequencing analysis.

### Preparation of everted membrane vesicles

For the assay of Na^+^/H^+^ antiport activity, *E. coli* KNabc cells carrying KNabc/pUC-UPF0118 or the empty vector pUC18 (as a negative control) were grown in LBK medium up to the mid-exponential phase and harvested by centrifugation at 5000 g, 4 °C for 10 min. Everted membrane vesicles were prepared by French pressure cell method at 2000 psi and collected by ultracentrifugation at 100, 000 g for 1 h as described by Rosen^45^. The vesicles were resuspended in a buffer containing 10 mM Tris-Cl (pH 7.5), 140 mM choline chloride, 0.5 mM dithiothreitol and 250 mM sucrose, and stored at −80 °C before use.

### Detection and localization of UPF0118 by western blot

For the detection and localization of UPF0118, *E. coli* KNabc carrying pET-22b-UPF0118 and the empty vector pET-22b (as a negative control) were grown in LBK medium to OD_600_ between 0.4 and 0.6 at 37 °C, followed by induction by the addition of isopropyl-β-D-thiogalactoside to a final concentration of 1 mM at 28 °C for an additional 6 h and then harvested by centrifugation at 5, 000 g, 4 °C for 10 min and washed three times with 10 mM Tris-HCl (pH 7.5). Cell pellets were frozen at −80 °C overnight to weaken the cell wall and resuspended in an ice-cold lysis buffer containing 50 mM Tris-Cl (pH 8.0), 2 mM EDTA, 100 mM NaCl and 0.1% Triton X-100. Cell suspension was lysed in the above mentioned ice-cold lysis buffer plus 1 mM PMSF, 1 mM DTT and 100 μg/ml lysozyme via an JY92-IIN ultrasonic cell mixer (NingBo Scientz Biotechnology Co., Ltd, China) in a pulsed mode (cycles: 4 sec ON followed by 5 sec OFF) until the lysate changed from an opaque solution into a less turbid solution. The lysed sample was centrifuged at 5,000 g at 4 °C for 10 min to remove large debris fragments and unlysed cells. A part of supernatant was sampled as a representative of the total protein extract including membrane and cytoplasmic protein fractions and the remain supernatant was ultracentrifuged at 100, 000 g for 1 h as described by Rosen^45^ to separate membrane protein fractions (pellets) from cytoplasmic protein ones (supernatant). SDS-PAGE and western blots were performed as described by Green *et al*.^46^. His_6_-tag detection was done using a rabbit anti-6× His-tag antibody (Abcom Ltd, China) and a goat anti-rabbit horseradish peroxidase-labeled secondary antibody (Nachuan Biotechnology Co., Ltd, Changchun, China). The BeyoECL Star kit (Beyotime Biotechnology Co. Ltd, China) was used and antibody binding was visualized by a Tanon-5200 multi chemiluminescence imaging system (Tanon Co. Ltd, China).

### Assay of Na^+^/H^+^ antiport activity

The Na^+^(Li^+^)/H^+^ antiport activity of everted membrane vesicles was estimated according to the extent of the collapse of a transmembrane proton gradient, with acridine orange as the pH indicator, as described by Rosen^45^. The assay mixture contained 10 mM BTP (BisTris-Propane) (at the indicated pH from 6.5 to 9.5), 140 mM choline chloride, 5 mM Mg_2_SO_4_, 1 μM acridine orange and membrane vesicles (equivalent of 40 μg ml^−1^ total protein). Tris-D-lactic acid (final concentration 10 mM) was added to initiate respiration. After the fluorescence quenching reached steady state, NaCl, LiCl or Na-free KCl with high purity (99.9995%, Sigma-Aldrich Co. LLC.) (To avoid the contamination of traces of NaCl) was added to the final concentration of 10 mM and then the fluorescence was dequenched. The ratio of fluorescence dequenching extent by NaCl or LiCl to the fluorescence quenching one by Tris-D-lactic acid was recorded as a representative of Na^+^(Li^+^)/H^+^ antiport activity. Fluorescence was monitored with a Hitachi F-7000 fluorescence spectrophotometer (Hitachi Ltd, Tokyo, Japan) at excitation and emission wavelength of 492 and 526 nm, respectively.

### The plotting of Michaelis-Menten kinetics of UPF0118 and calculation of the apparent *K*_*m*_ and *V*_*max*_ values

For the plotting of Michaelis-Menten kinetics of UPF0118, pH was adjusted to 9.0 based on the highest Na^+^/H^+^ and Li^+^/H^+^ antiport activity, and the different Na^+^ or Li^+^ concentrations were varied from 0 to 15 mM. Fluorescence dequenching percentages at the corresponding cation concentrations were recorded as their respective representatives of Na^+^(Li^+^)/H^+^ antiport activity. The Michaelis-Menten kinetics of UPF0118 was plotted with Na^+^/H^+^ and Li^+^/H^+^ antiport activity as the respective function of cation concentrations. For the calculation of the apparent *K*_*m*_ and *V*_*max*_ values of UPF0118, non-linear regression analysis was carried out and the corresponding apparent *K*_*m*_ and *V*_*max*_ values were obtained with Prism 5.0.

### DNA manipulation and sequence analysis

Preparation of plasmid DNA, extraction of genomic DNA, restriction enzyme digestion and ligation were carried out as described by Green *et al*.^46^. DNA sequencing was performed by Beijing Genomics Institute (Beijing, China). The analyses for ORF and hydrophobicity were carried out with the DNAMAN 6.0 software. Protein sequence alignment was performed through the National Center for Biotechnology Information (NCBI) using the website http://www.ncbi.nlm.nih.gov/blastp43. A phylogenetic tree including UPF0118 and its homologs, together with all known specific single-gene Na^+^(Li^+^)/H^+^ antiporters and single-gene proteins with the Na^+^(Li^+^)/H^+^ antiport activity, was constructed by using the neighbour-joining method^47^. The stability of clusters was ascertained by performing a bootstrap analysis (1000 replications). Promoter prediction was performed using the website http://www.fruitfly.org/seq_tools/promoter.html. Transmembrane segment prediction was carried out by using the website http://mobyle.pasteur.fr/cgi-bin/portal.py?#forms::toppred 48.

### Protein content determination

Protein concentration in everted membrane vesicles was determined by the method of Lowry *et al*.^49^ with bovine serum albumin as a standard.

### Nucleotide sequence accession number

The nucleotide sequence of UPF0118 reported in this paper has been submitted to GenBank database with the accession number KY231907.

## Additional Information

**How to cite this article**: Dong, P. *et al*. A UPF0118 family protein with uncharacterized function from the moderate halophile *Halobacillus andaensis* represents a novel class of Na^+^(Li^+^)/H^+^ antiporter. *Sci. Rep.* **7**, 45936; doi: 10.1038/srep45936 (2017).

**Publisher's note:** Springer Nature remains neutral with regard to jurisdictional claims in published maps and institutional affiliations.

## Supplementary Material

Supplementary Material
