# Supplementary material for: A UPF0118 family protein with uncharacterized function from the moderate halophile Halobacillus andaensis represents a novel class of Na+(Li+)/H+ antiporter
Source: Sci Rep. 2017 Apr 4;7:45936. doi: 10.1038/srep45936 (PMC5379678; doi:10.1038/srep45936)
Supplement: Supplementary Material [file srep45936-s1.pdf]

Supplemental Material

A UPF0118 family protein with uncharacterized function from the moderate halophile *Halobacillus andaensis* represents a novel class of $Na^+(Li^+)/H^+$ antiporter

Ping Dong, Lidan Wang, Na Song, Lina Yang, Jin Chen, Mingxue Yan, Huiwen Chen, Rui Zhang, Jincheng Li, Heba Abdel-motaal, Juquan Jiang*

Department of Microbiology and Biotechnology, College of Life Sciences, Northeast Agricultural University, Harbin, 150030, PR China

* Authors for correspondence: Juquan Jiang, Tel: 86-451-55191132. Fax: 86-451-55190413. E-mail address: jjqdainty@163.com.

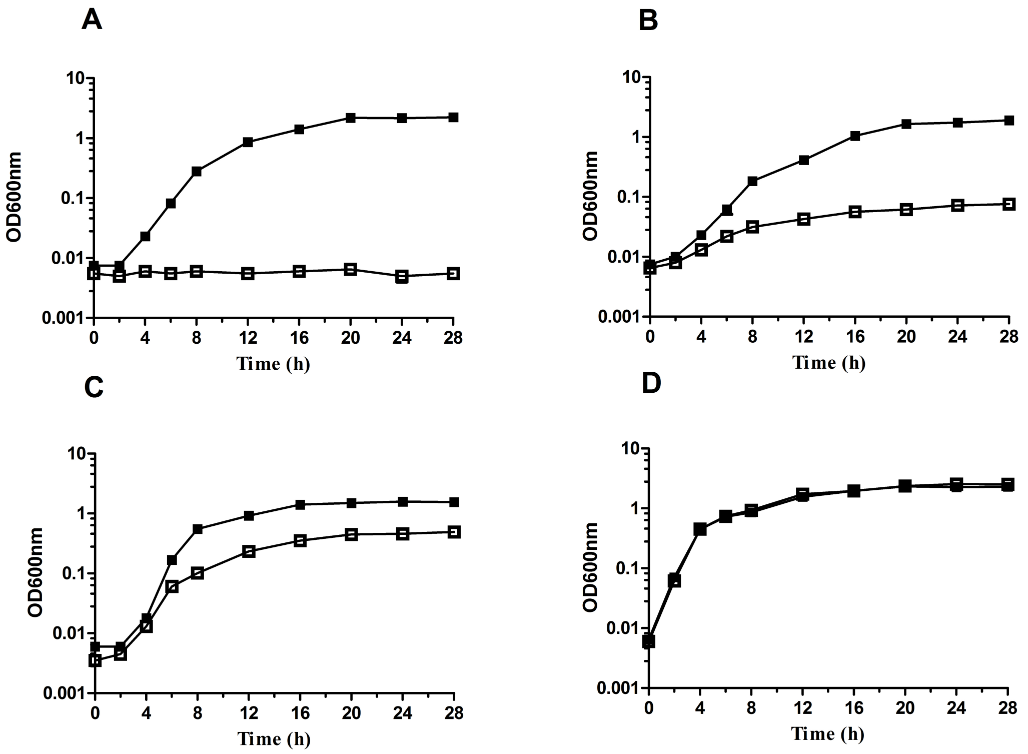

Fig. S1. Growth curves of *Escherichia coli* KNabc under the saline and alkaline conditions.

For the more detailed analysis of the resistance of UPF0118 to NaCl, LiCl and pH, 1% overnight cultures of KNabc cells carrying the empty vector pUC18 (open square) or pUC-UPF0118 (filled square) grown at 37 ºC in the LBK medium at pH 7.0 were innoculated into fresh LBK medium at pH 7.0, to which 0. 2 M NaCl (A) or 5 mM LiCl (B) was added, or fresh LBK medium containing 50 mM NaCl at pH 7.5 (C) adjusted by adding the Hepes-Tris buffer at the final concentration of 100 mM, followed by incubation at 37 ºC. Also, to show cell growth in the absence of the salts at neutral pH, the KNabc transformant cells carrying the empty vector pUC18 (open square) or pUC-UPF0118 (filled square) grown at 37 ºC in the LBK medium at pH 7.0 were innoculated into fresh LBK medium without the addition of the tested salts at pH 7.0 (D) adjusted by adding the Hepes-Tris buffer at the final concentration of 100 mM, followed by incubation at 37 ºC. The above mentioned cell growth was monitored turbidimetrically at 600 nm at the indicated time points within 28 h. Each data point

represents the average of three independent determinations. The growth cuvres were plotted on a semilogarithmic scale.
